# *Saccharomyces Boulardii* Ameliorates Non-alcoholic Steatohepatitis in Mice Induced by a Methionine-Choline-Deficient Diet Through Gut-Liver Axis

**DOI:** 10.3389/fmicb.2022.887728

**Published:** 2022-06-23

**Authors:** An-Ming Yang, Chien-Yu Lin, Shih-Hao Liu, Guan-Da Syu, Hao-Jhe Sun, Kuei-Chuan Lee, Han-Chieh Lin, Ming-Chih Hou

**Affiliations:** ^1^Department of Internal Medicine, En Chu Kong Hospital, New Taipei City, Taiwan; ^2^Department of Nursing, Yuanpei University of Medical Technology, Hsinchu, Taiwan; ^3^School of Medicine, Fu Jen Catholic University, New Taipei City, Taiwan; ^4^Division of Pathology, En Chu Kong Hospital, New Taipei City, Taiwan; ^5^Department of Biotechnology and Bioindustry Sciences, National Cheng Kung University, Tainan, Taiwan; ^6^International Center for Wound Repair and Regeneration, National Cheng Kung University, Tainan, Taiwan; ^7^Department of Medicine, National Yang Ming Chiao Tung University School of Medicine, Taipei City, Taiwan; ^8^Division of Gastroenterology and Hepatology, Department of Medicine, Taipei Veterans General Hospital, Taipei City, Taiwan

**Keywords:** non-alcoholic steatohepatitis, intestinal commensal fungi, *Saccharomyces boulardii*, methionine-choline deficiency diet, gut-liver axis

## Abstract

Non-alcoholic steatohepatitis (NASH) is affecting people worldwide. Changes in the intestinal microbiome are crucial to NASH. A previous study showed that eradicating intestinal fungi ameliorates NASH; however, the role of intestinal fungi in the development of NASH remains unclear. *Saccharomyces boulardii (SB)*, a dietary supplement yeast, has been reported to restore the integrity of the intestine. Here, we tested the effect of *SB* in the treatment of NASH. For this study, we fed eight-week-old C57/BL6 male mice either a methionine-choline deficient (MCD) diet or a normal chow diet (NCD) for eight weeks. Half of the MCD diet-fed mice were gavaged with SB (5 mg/day) once daily. The remainder of the NCD–fed mice were gavaged with normal saline as a control. The MCD diet-fed mice on *SB* supplement showed better liver function, less hepatic steatosis, and decreased inflammation. Both hepatic inflammatory gene expression and fibrogenic gene expression were suppressed in mice with *SB* gavage. Intestinal damage caused by the MCD diet was tampered with, intestine inflammation decreased, and gut permeability improved in mice that had been given the *SB* supplement. Deep sequencing of the fecal microbiome showed a potentially increased beneficial gut microbiota and increased microbiota diversity in the *SB*-supplemented mice. The *SB* supplement maintains gut integrity, increases microbial diversity, and increases the number of potentially beneficial gut microbiota. Thus, the *SB* supplement attenuates gut leakage and exerts a protective effect against NASH. Our results provide new insight into the prevention of NASH.

## Introduction

Non-alcoholic fatty liver disease (NAFLD) affects many people worldwide. The global prevalence of this disease is 25.24%. Metabolic comorbidities such as type 2 diabetes, hyperlipidemia, hypertension, and obesity are associated with NAFLD (Younossi et al., 2016). The spectrum of NAFLD ranges from simple steatosis to non-alcoholic steatohepatitis, resulting in liver fibrosis, cirrhosis, and even hepatocellular carcinoma (le Roy et al., 2013). The causes of NAFLD are multifactorial, including genetic, lifestyle, dietary, and environmental factors. In view of the fact that NAFLD has a high prevalence and is associated with significant comorbidities, extensive research has been conducted in recent decades for potential treatment. Although dozens of clinical trials targeting different mechanisms are ongoing, no definite agent has been approved by the US Food and Drug Administration (FDA) (Vuppalanchi et al., 2021).

Fatty acids in the liver, which come from both excess dietary fat and increased *de novo* hepatic lipogenesis, may cause NAFLD and NASH (Argo and Caldwell, 2009). Abnormal accumulation of fatty acids in the liver may trigger a subsequent inflammatory response, which has been defined as lipotoxicity (Malhi and Gores, 2008). Previous research has revealed that infiltrating innate immune cells in the sinusoids of the liver may trigger inflammatory responses that result in NASH (Chen et al., 2020). These innate immune cells express elevated levels of pathogen recognition receptors (PRR), which recognize both pathogen-associated molecular patterns (PAMPs) from the portal circulation and damage-associated molecular patterns (DAMPs) due to the lipotoxicity inside the liver (Arrese et al., 2016). Recent studies have shown that intestinal dysbiosis leads to increased endotoxin in the portal vein, causing inflammation in the liver (Schnabl, 2013). In addition, NASH is associated with increased intestinal permeability, which indicates that an early phase of liver injury and inflammation contributes to the latter (Luther et al., 2015). Furthermore, changes in human gut microbiota have been reported to be associated with human body fat composition and gut permeability (Schneider et al., 2015; Baothman et al., 2016). This evidence indicates a “gut-liver cross-talk” pattern in the pathogenesis of NAFLD and NASH. Many studies have confirmed the importance of intestinal microorganisms in liver disease (Schnabl and Brenner, 2014). The relationship between intestinal commensal bacteria and liver disease has been well established. However, only a few studies focused on the relationship between intestinal commensal fungi and liver disease. Yang et al. demonstrated that commensal fungi contribute to alcoholic liver disease (Yang et al., 2017). However, the relationship between intestinal fungi and NAFLD is still not clear. Additionally, the interaction between intestinal commensal bacteria and fungi is unknown. A commercialized yeast, *Saccharomyces boulardii* (*SB*), often used as a probiotic in a lyophilized form to treat diarrhea, has been reported to restore the integrity of the intestine and attenuate diarrhetic leakage (Stier and Bischoff, 2016). *SB* has been reported to reduce carbon tetrachloride-caused liver fibrosis (Li et al., 2015) and liver injury caused by Salmonella enteritis (Wu et al., 2014). *SB* is a beneficial aerobic fungus that could regulate intestinal microbial homeostasis, interfere with the ability of pathogens to colonize and infect the mucosa, and modulate local and systemic immune responses (Kelesidis and Pothoulakis, 2012). Everard et al. (2014) reported that oral administration of *SB* reduced bodyweight, fat mass, hepatic steatosis, and inflammation in obese and type 2 diabetic db/db mice. However, there is no evidence of the effect of *SB* in the NASH mouse model. Methionine-choline-deficient (MCD) diet feeding results in steatosis, inflammation, fibrosis in the liver and shortening intestinal villi with disruption of small intestinal tight junctions (Kawauchi et al., 2019). Moreover, the intestinal microbiota can protect against MCD diet-caused liver injury (Schneider et al., 2019). Therefore, we aimed to investigate the possible protective effect of *SB* on the gut-liver axis in the MCD diet-fed mice.

## Experimental Section

### Animal

Animal experiments were supervised and approved by the Institutional Animal Care and Use Committee of Taipei Veterans General Hospital, Taiwan (IACUC number: 2018–012). Male wild type C57BL/6J littermate mice purchased from the National Laboratory Animal Center of Taiwan were kept in filter-toped cages. Mice after eight weeks were included in these experiments. The MCD diet (Mod TestDiet^®^ 5CC7 w/no added choline and methionine) was fed for eight weeks to induce liver injury (*n* = 19). Further, a normal chow diet (NCD) (*n* = 10) was used as the control diet.

To test the effect of *SB*, we randomly administered 5 mg of *SB* to mice on the MCD diet or NCD 5 mg of *SB* (Florastor^®^, Biocodex, France; dispended in 300 μl of ddH2O) by gavage five days a week (Saturday and Sunday off) (MCD–*SB*, *n* = 10; NCD–*SB*, *n* = 5) or the same amount of ddH2O (MCD–vehicle, *n* = 9; NCD–vehicle, *n* = 5). Florastor contains the active probiotic strain *Saccharomyces boulardii* lyo CNCM I-745. There are five billion CFU of *Saccharomyces boulardii* lyo CNCM I-745 in every 250 mg of Florastor. Assuming the α is 0.05 and the effect size is 1.6, the sample size with 1:2 allocation was calculated by G Power software to achieve a power of 0.8. After eight weeks of being fed with gavage, the mice were euthanized and sacrificed for subsequent experiments.

### Real-Time Quantitative Polymerase Chain Reaction

The RNA of mouse tissues was extracted by RNAzol^®^ RT (Sigma-Aldrich). The DNase treatment of RNA was performed using the DNA-free kit (Ambion), and RNA was reversely transcribed using the High-Capacity cDNA Reverse Transcription kit (ABI). Real-time quantitative polymerase chain reaction (qPCR) was performed with the PerfeCTa SYBR Green FastMix (QuantaBio) using primer sequences as shown in Supplementary Table 1 and a QuantStudio 3 (Thermo). The qPCR values were normalized to mouse 18S. The gene expression results are expressed relative to the levels of the NCD–vehicle group.

### Fecal DNA Extraction

Using the AccuPure Stool DNA manual kit (D24096, AccuBioMed, Taiwan), mouse fecal genomic DNA was extracted.

### Sequencing and Analysis

Metagenomic sequencing was performed on mouse fecal DNA, targeting the hypervariable V3 and V4 region of prokaryotic 16S rRNA loci. To prepare the library, we amplified the V3 and V4 regions using a limited cycle PCR and added Illumina sequencing adapters and dual-index barcodes to the amplicon target. To generate the sequence of MiSeq – using paired 300-bp reads and MiSeq v3 reagents, we overlapped the ends of each read to generate high-quality, full-length reads of the V3 and V4 regions in a single 65-h run. Amplicon sequencing was performed using 300-bp paired-end raw reads, and the entire paired-end reads were assembled using FLASH v.1.2.7 (Magoč and Salzberg, 2011). De-multiplexing was carried out based on barcode identification. To analyze the sequence similarities among different OTUs, multiple sequence alignment was conducted by using the PyNAST software (v.1.2) (Caporaso et al., 2010) against the core-set dataset in the Silva database. To normalize the variations in sequence depth across samples, OTU abundance information was rarefied to the minimum sequence depth using the QIIME script (single_rarefaction.py, the minimum effective tags was 29856). Subsequent analyses of alpha and beta diversities were both performed using the normalized data. Alpha diversity was indicative of the species complexity within individual samples based on seven different criteria output from the QIIME pipeline (Schloss et al., 2009). Beta diversity analysis was performed to evaluate the differences in terms of species complexity among samples. One beta diversity parameter, weighted UniFrac (Lozupone and Knight, 2005; Lozupone et al., 2011), was calculated using the QIIME pipeline. A cluster analysis was preceded by a principal component analysis (PCA), which was applied to reduce the dimensions of the multiple variables using the FactoMineR package and ggplot2 package in R software (v.2.15.3). Principal Coordinate Analysis (PCoA) was performed using the distance matrix to acquire principal coordinates for the visualization of sophisticated and multidimensional data (Jiang et al., 2013). Statistically significant biomarkers were identified using the LEfSe analysis (Segata et al., 2011). In brief, LEfSe is an approach based on an algorithm that performs the non-parametric Kruskal–Wallis test and Wilcoxon rank-sum test to identify bacterial taxa whose relative abundance is a significant difference between the control and interest. LEfSe applies LDA to those bacterial taxa identified as significantly different and further assesses the effect size of each differentially abundant taxon. In this study, taxa with an LDA score (log 10) > 4 were considered significant.

To evaluate mouse intestinal fungi, metagenomic sequencing targeting the eukaryote 18S rRNA ITS1/ITS2 region was performed on mouse fecal DNA. The distinct regions were amplified using specific primers (ITS1: ITS5-1737F GGAAG TAAAAGTCGTAACAAGG, ITS2-2043R GCTGCGTTCTTCA TCGATGC; ITS2: ITS3-2024F GCATCGATGAAGAACGCA GC, ITS4-2409R TCCTCCGCTTATTGATATGC) with the barcode. All PCR reactions were carried out with Phusion^®^ High-Fidelity PCR Master Mix (New England Biolabs). The sequencing libraries were generated using TruSeq^®^ DNA PCR-Free Sample Preparation Kit (Illumina, United States) following the recommendations of the manufacturer, and index codes were added. Additionally, the non-ITS sequence was removed. The library quality was assessed on the Qubit@ 2.0 Fluorometer (Thermo Scientific) and the Agilent Bioanalyzer 2100 system. Finally, the library was sequenced on an Illumina NovaSeq6000 platform, and 250 bp paired-end reads were generated. The bioinformatics analysis was similar to 16S, except that the sequence alignment was performed against the NCBI database. The sequence data were registered at NCBI under BioProject PRJNA817530.

### Protein and Biochemical Analysis

Fecal albumin levels were measured by ELISA (Mouse Albumin ELISA kit E99-134, BETHYL). The plasma levels of alanine aminotransferase (ALT) and hepatic levels of triglyceride were measured using the Infinity ALT kit (Thermo Fisher Scientific) and Triglyceride Liquid Reagents Set (T7532-120, Pointe Scientific), respectively. Plasma lipopolysaccharide (LPS) levels were measured by ELISA (CEB526Ge; Cloud-Clone Corp.).

### Staining Procedures

The formalin-fixed liver sections were stained with H & E, Sirius red, and Masson’s trichrome to detect any liver injury and fibrosis. Further, intestine sections were stained with H & E. The liver HE-stained sections were scored using the NAFLD activity score (NAS) system (Brunt et al., 2011) by our co-author H.-S.L.—an experienced pathologist—and the degree of fibrosis was evaluated by Sirius red stain (Picrosirius Red Stain Kit, Polysciences, Inc., Warrington, PA, United States) and Masson’s trichrome staining (Muto pure chemical co., Ltd.). NIH ImageJ was used to estimate the length and width of gut villi and crypts.

Paraffin-embedded liver sections were stained for F4/80 (ab6640, abcam^®^) and stained for alpha-smooth actin (a-SMA) (GTX100034, GeneTex). Paraffin-embedded intestine sections were stained for ZO-1 (NBP1-85047, Novus). Standard immunohistochemical (IHC) staining procedures were employed; liver and intestine sections were incubated with a specific primary antibody, as described earlier, followed by incubation with an HRP-linked secondary antibody (Dako, Glostrup, and Denmark) and 3,30-diaminobenzidine (Dako, Glostrup, and Denmark) and were scanned using the NanoZoomer Digital Pathology system.

### Statistical Analysis

The results were expressed as mean ± SEM, and the significance was evaluated using the unpaired Student’s *t*-test, except when stated otherwise. The significance of mouse microbiome data was determined using the non-parametric Kruskal–Wallis test and Wilcoxon rank-sum test. A *p*-value less than 0.05 was considered to be statistically significant.

## Results

### *Saccharomyces boulardii* Alleviated Methionine-Choline Deficient Diet-Caused Hepatic Steatosis and Injury

**FIGURE 1:**
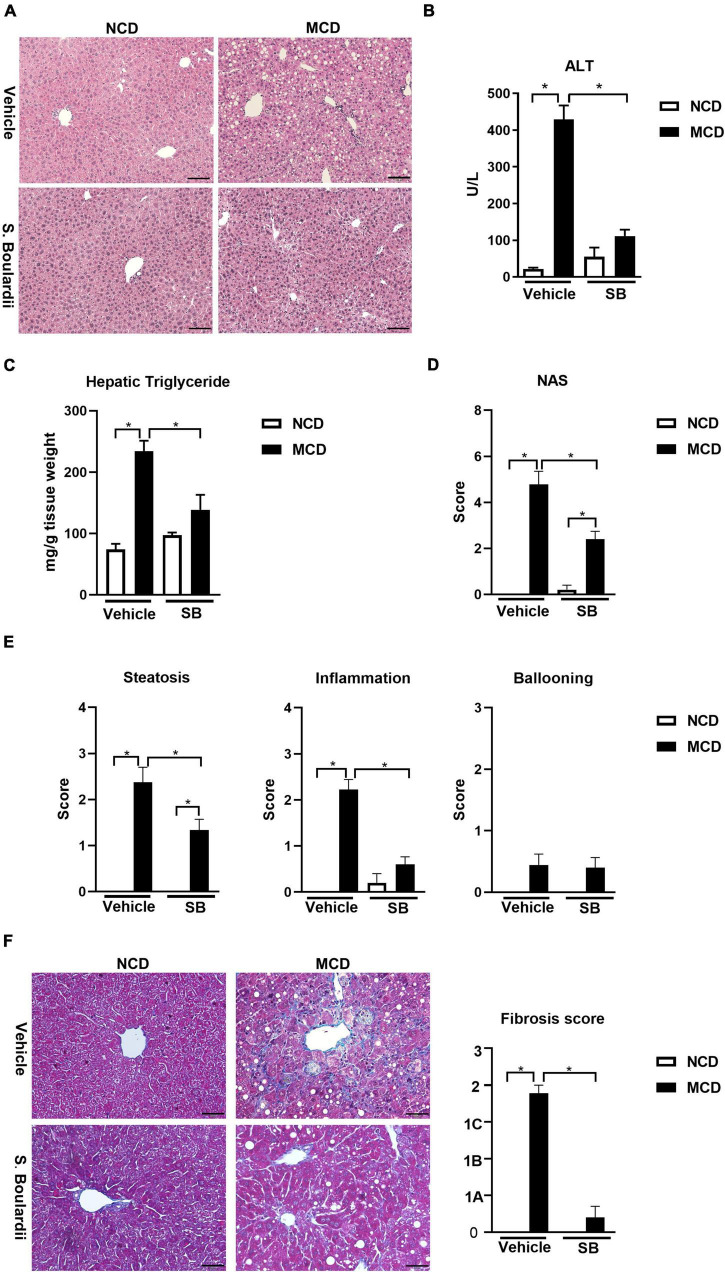
*SB* reduced MCD diet-caused steatohepatitis in mice. C57BL/6 male mice were fed an oral control diet (*n* = 10) or the MCD diet (*n* = 18) and given vehicle or *SB*. **(A)** Representative liver sections after H & E staining, scale bars: 100μm. **(B)** Plasma levels of alanine aminotransferase (ALT). **(C)** Hepatic triglyceride content. **(D)** NAFLD activity score (NAS). **(E)** Parameters of NAS: Steatosis, inflammation and ballooning. **(F)** Representative sections of Trichrome Masson staining, scale bar: 50μm, and fibrosis score based on the NAS scoring system. *p < 0.05.

The livers of the mice on the MCD diet presented more extensive fat accumulation than those in the NCD groups. Interestingly, the mice on the MCD diet with *SB* administration showed markedly less fat accumulation, which was close to the NCD–vehicle group (Figure 1A). The plasma alanine-transaminase (ALT) levels were significantly lower in the MCD–*SB* group than in the MCD–vehicle group (Figure 1B). Liver triglyceride levels in the MCD–SB group were significantly lower than those in the MCD–vehicle group (Figure 1C). The liver-to-bodyweight ratio of the MCD–vehicle group was significantly lower than that in the NCD–vehicle group; however, in the *SB* gavage groups, the liver-to-body weight ratio became insignificant (Supplementary Figure 1A). The bodyweight of mice on the MCD diet with or without *SB* was unchanged, and so were those in the NCD groups (Supplementary Figure 1B).

The liver sections of this cohort were reviewed by a widely used scoring system— “NAFLD activity score (NAS)” (Brunt et al., 2011). We found that the MCD diet-fed mice on *SB* showed significantly less steatosis than those with the MCD diet and vehicles (Figure 1E). Furthermore, the inflammation scores of the mice on the MCD diet plus *SB* gavage almost returned to the degree of the NCD–vehicle group (Figure 1E). Although the ballooning scores did not differ between the four groups, the NAS was significantly lower in the MCD–*SB* group compared to the MCD–vehicle group (Figure 1D). For the fibrosis score mentioned in the NAS scoring system, Trichrome-Masson staining of the MCD diet-fed mice showed marked fibrosis; it was significantly lower than in the MCD–*SB* group (Figure 1F). These findings indicate that *SB* could ameliorate hepatocellular injury and steatosis caused by the MCD diet.

### *Saccharomyces boulardii* Ameliorated Hepatic Inflammation With Less Kupffer Cell Activation

**FIGURE 2:**
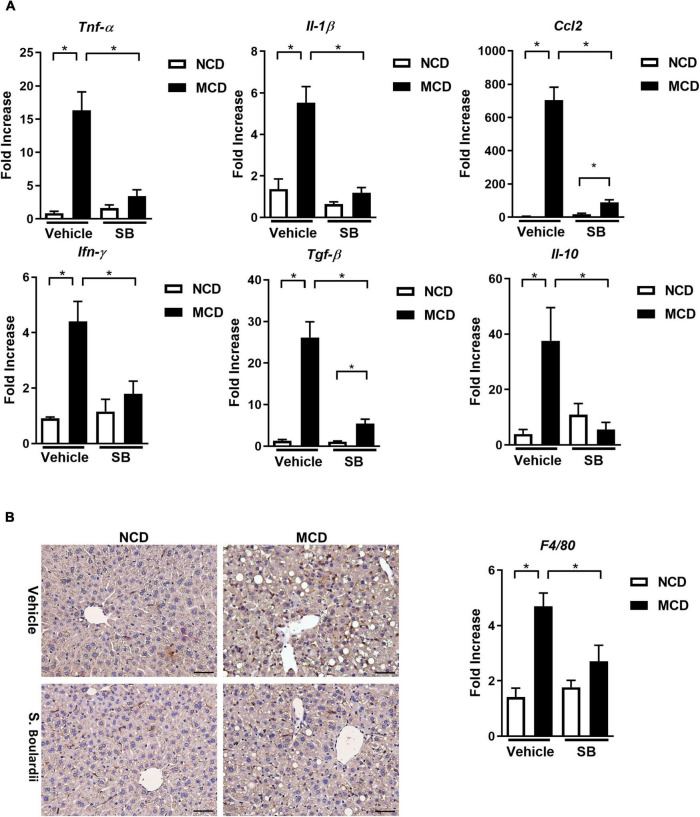
Hepatic inflammatory responses caused by the MCD diet were suppressed by *SB* gavage. **(A)** Hepatic expressions of proinflammatory cytokines. **(B)** Representative liver sections of immunohistochemical staining of F4/80 and hepatic expression of F4/80. Scale bars: 50μm. *p < 0.05.

Tumor necrosis factor-alpha (TNF-α), the acute phase reaction cytokine produced by macrophages, was caused in the MCD diet-fed mice and lowered in the MCD–*SB* group. Similarly, IL-1β, a cytokine secreted by macrophages and regulated by the Caspase-1 inflammasome, also showed a similar alternation (Figure 2A). These findings implied that *SB* gavage could reduce the acute phase inflammatory reaction caused by macrophages and Kupffer cells in the livers of the MCD diet-fed mice.

Hepatic CCL-2 expression was raised in the MCD diet-fed mice, which was reduced in the MCD diet-fed mice gavage by *SB*. It means that *SB* could decrease the chemotactic effect and recruit fewer inflammatory cells in the MCD diet-fed mice. The hepatic gene expression of INF-γ and TGF-β also showed similar patterns. Interestingly, the anti-inflammatory cytokine, IL-10, was also induced in the MCD diet-fed mice and decreased in the MCD diet-fed mice on *SB* gavage; this phenomenon could be a less compensatory response of IL-10 to less inflammation in the MCD–*SB* group (Figure 2A). F4/80 expression was markedly induced in the MCD diet-fed mice and was suppressed in the MCD–*SB* group (Figure 2B). These results showed that *SB* gavage in the MCD diet-fed mice suppressed the inflammatory responses in the liver with less Kupffer cell activation.

### Methionine-Choline Deficient Diet-Caused Hepatic Fibrosis Was Reduced in Mice on the *Saccharomyces boulardii* Supplement

**FIGURE 3:**
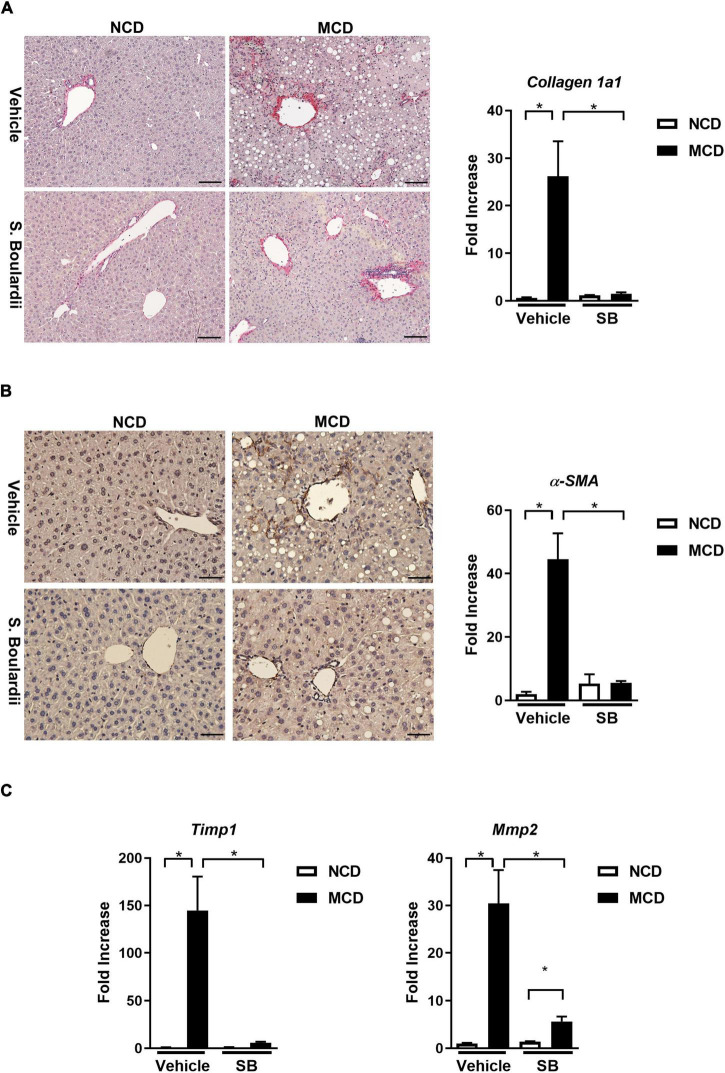
SB ameliorated MCD diet-caused hepatic fibrosis in mice. **(A)** Representative sections of Sirus Red staining (Scale bar: 100μm) and hepatic c*ollagen 1a1* expression. **(B)** Representative sections of immunohistochemical staining ofα-SMA (Scale bar: 50μm) and hepatic expression ofα-*SMA.* **(C)** Hepatic fibrotic gene expressions, Tissue Inhibitor of Metalloproteinase 1 (*Timp1)* and Matrix Metallopeptidase 2 (*Mmp2)* *p < 0.05.

Following the results of changes in inflammation, we evaluated the degree of fibrosis in this cohort. Interestingly, the hepatic gene expression levels of collagen 1a1 were almost restored to normal levels in the MCD–*SB* group. Also, the liver section on Sirius red staining showed a significantly reduced positive area in the MCD–*SB* group (Figure 3A). Immunohistochemical staining of α-SMA on the liver sections also showed a less positive area in the MCD–*SB* group as well as the hepatic gene expression (Figure 3B). The hepatic gene expression of *Timp1* and *MMP2* was restored to baseline levels in the MCD–*SB* group (Figure 3C). Our results showed *SB* gavage could significantly reduce MCD diet-caused hepatic fibrosis in mice. This effect may probably be due to reduced inflammation in the liver.

### *Saccharomyces boulardii* Prevented the Intestine Injury Caused by the Methionine-Choline Deficient Diet

**FIGURE 4:**
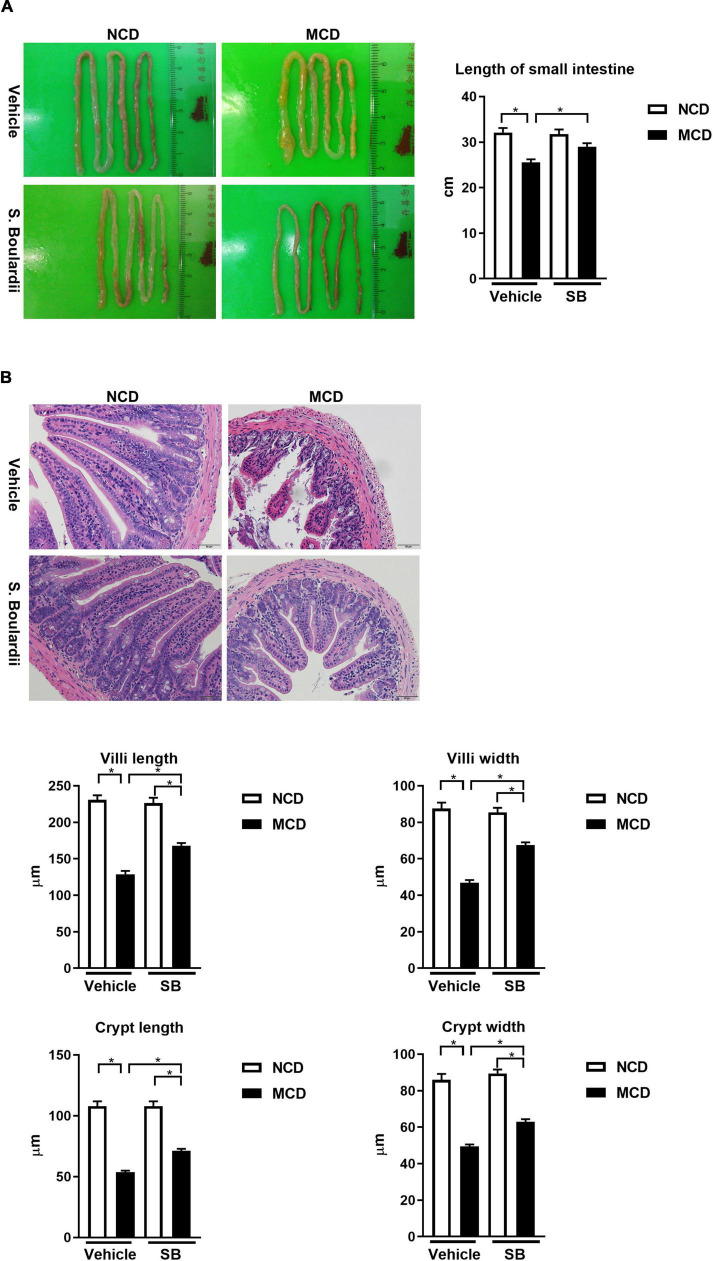
The MCD diet led to the shortening of the small intestine and intestinal villi, which were improved by the *SB* supplement. **(A)** Representative images of the small intestine of mice on the control diet (*n* = 10) or the MCD diet (*n* = 18), and also given vehicle or *SB*. The length of the small intestine improved after the *SB* supplement. **(B)** Representative sections of the distal small intestine after H & E staining. Both the intestine length of villi and the width of crypt improved after the *SB* supplement. Scale bars: 50μm. *p < 0.05.

It has been reported that MCD diet feeding could result in shortening of the small intestine and atrophy of the small intestinal villi (Schneider et al., 2019). The MCD diet-fed mice had consistently shorter small intestines than the NCD–vehicle group (*p* < 0.001). Interestingly, this phenomenon was not seen in the MCD–SB group (Figure 4A).

We next evaluated the effect of *SB* on the villi using the sections with H & E staining of the distal small intestine of the mice (Figure 4B). The MCD diet-fed mice showed an atrophic change of the intestinal villi with narrower crypts. In the MCD–*SB* group, both intestinal villi length and crypt width become significantly longer and wider than in the MCD–vehicle group (Figure 4B). These results indicated that *SB* could prevent intestinal injury caused by the MCD diet in mice.

### Methionine-Choline Deficient Diet-Fed Mice on *Saccharomyces boulardii* Showed Less Inflammation and Less Leakage in the Intestine

**FIGURE 5:**
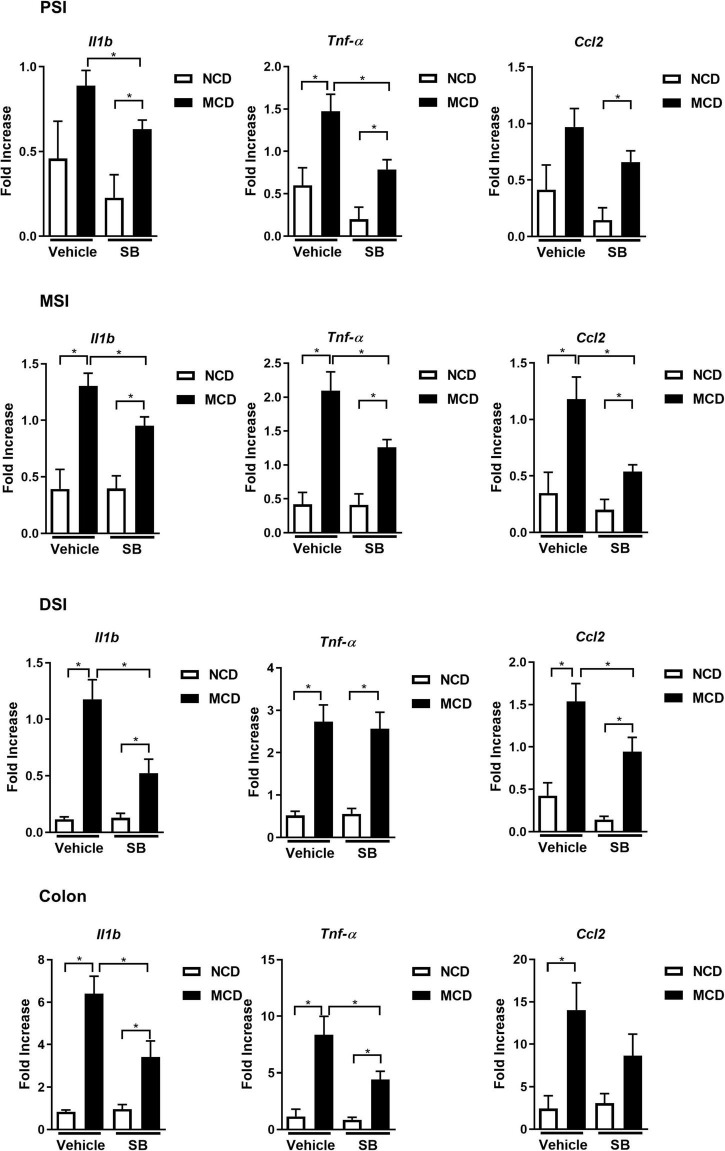
SB ameliorated the MCD diet-caused intestinal inflammation. Intestinal expression of proinflammatory cytokines *Il1b, Tnf*-α, and *CCL2* in the proximal, middle, distal small intestine (PSI, MSI, and DSI), and colon. *p < 0.05.

The MCD–vehicle mice exhibited significantly higher IL-1β levels in the proximal, middle, and distal small intestine, and *SB* supplementation decreased IL-1β levels in the *SB* and MCD diet-fed mice. The expression of acute reaction cytokine, TNF-α, was also markedly induced by MCD diet feeding; however, it was only suppressed in the SB gavage group in the proximal and middle intestine, not the distal intestine. In contrast, CCL2, which represents chemotaxis, was only suppressed in the middle and distal small intestine of the *SB* gavage group. The proinflammatory cytokine gene expression in the colon was the same as that of the proximal small intestine—SB suppressed IL1-β and TNF-α, but not CCL2 (Figure 5). These results showed *SB* could suppress, albeit not completely, the inflammation in the intestine caused by the MCD diet in mice. The reason for different inflammatory suppression patterns in different parts of the intestine merits further research.

**FIGURE 6:**
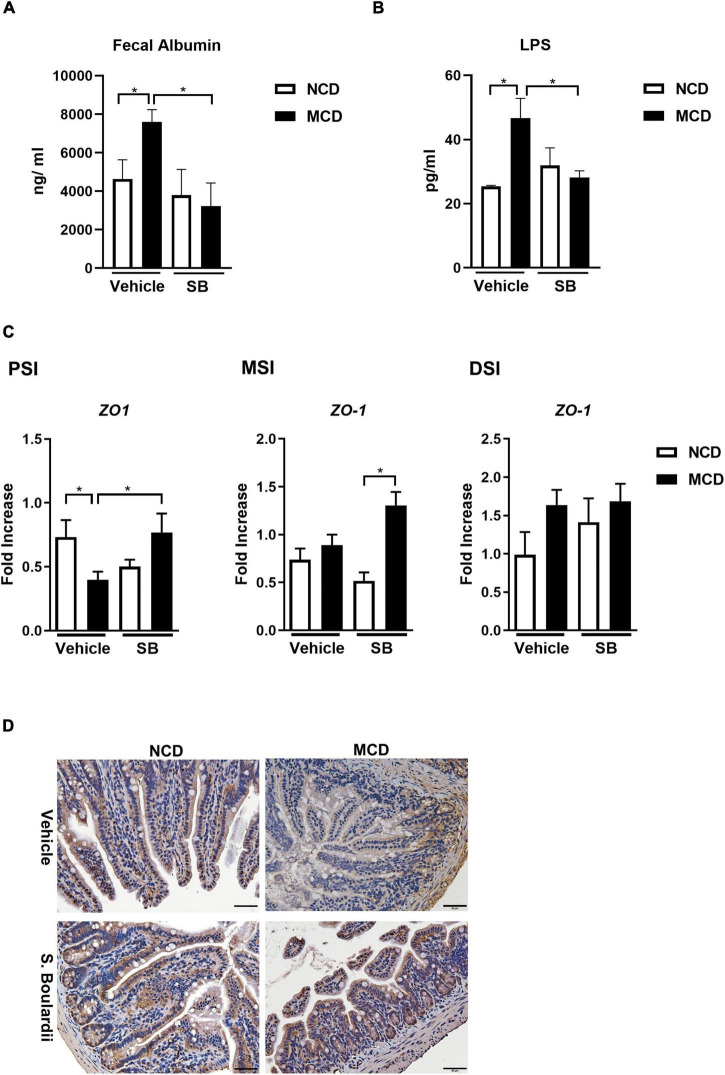
SB decreased the MCD diet-caused leaky gut with less Lipopolysaccharide translocation. **(A)** Fecal albumin measured by ELISA. **(B)** Plasma Lipopolysaccharide (LPS) level. **(C)** Intestinal expression of intestinal barrier gene, *ZO-1*, in the proximal, middle and distal small intestine. **(D)** Representative liver sections of ZO-1 immunohistochemical staining of the distal small intestine. Scale bars: 50μm. *p < 0.05.

We further evaluated the integrity of the intestine. The overall intestinal permeability was evaluated by fecal albumin, which was increased in the MCD diet-fed mice and returned to baseline in the *SB* gavage group, suggesting that *SB* gavage could prevent MCD diet-related leaky gut (Figure 6A). Moreover, plasma LPS levels showed the same pattern as fecal albumin levels, indicating that *SB* gavage prevents endotoxin translocation to portal circulation (Figure 6B). Tight junction protein ZO-1 gene expression was suppressed in the MCD diet-fed mice only in the proximal small intestine and could be reserved by *SB* gavage. Notably, in the middle small intestine, ZO-1 was not suppressed by the MCD diet but had higher gene expression in the SB gavage group (Figure 6C). Immunohistochemical staining of ZO-1 on the distal small intestine showed decreased ZO-1 protein in the intestinal section of the MCD diet-fed mice and showed nearly normal in mice on *SB* (Figure 6D).

Taking these findings together, we found that *SB* gavage in MCD diet-fed mice led to less intestinal inflammation, better gut integrity, and less leaky gut, which could interrupt the vicious circle in the gut-liver axis caused by the MCD diet, leading to minor liver injury.

### *Saccharomyces boulardii* Affected the Gut Microbial Configuration and Reserved Intestinal Microbiota Diversity in the Methionine-Choline Deficient Diet-Fed Mice

To obtain further insight into the protective effect of *SB*, we investigated the impact of *SB* on the gut microbiome.

**FIGURE 7:**
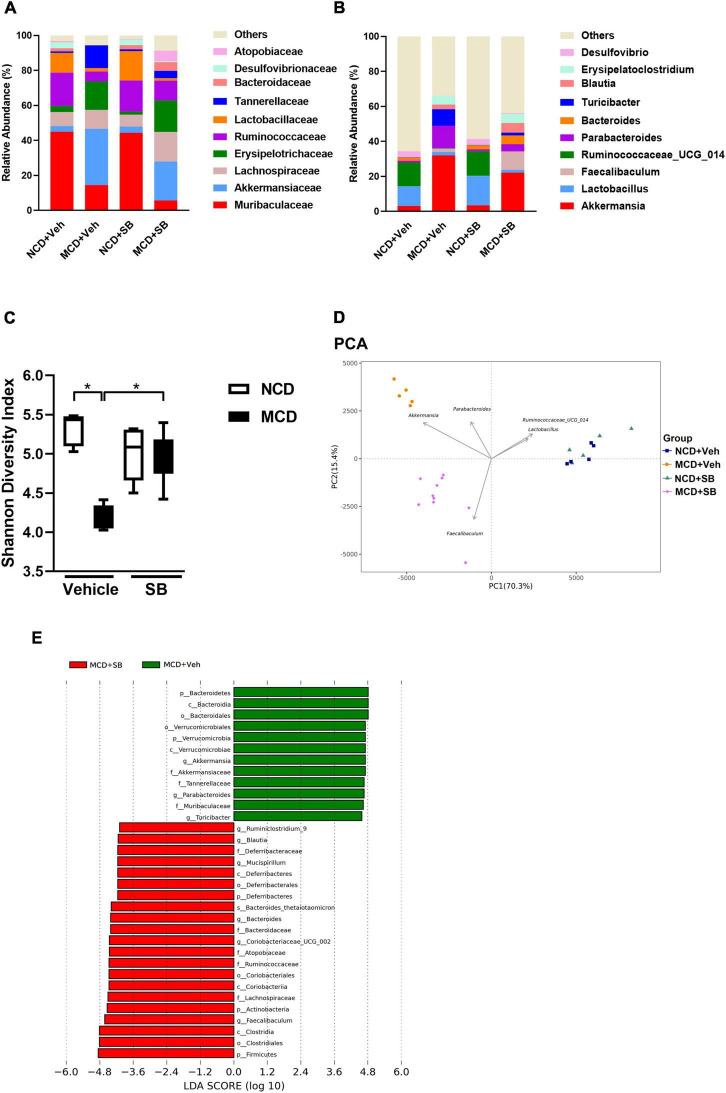
SB affected the microbial configuration and restored intestinal microbiota diversity in the MCD diet-fed mice. **(A)** Topmost abundant taxa at the family level and **(B)** genus level. **(C)** The Shannon diversity index represents alpha diversity. **(D)** PCA represents beta diversity at the genus level. Each symbol represents one sample (*n* = 5–10 per group). **(E)** Discriminative biomarkers with an LDA score > 4.0.

At the family level, the MCD–vehicle group had more *Akkermansiaceae, Erysipelotrichaceae*, and *Tannerellaceae* (*p* < 0.01) than the NCD–vehicle group, whereas *Muribaculaceae, Ruminococcaceae*, and *Lactobacillaceae* (*p* < 0.01) were more abundant in the NCD–vehicle group. Among the MCD–*SB* mice, *Lachnospiraceae, Atopobiaceae*, and *Ruminococcaceae* were more abundant (*p* < 0.01) (Figure 7A and Supplementary Figures 2A, 3A).

At the genus level, *Akkermansia*, *Parabacteroides*, and *Turicibacter* were significantly more abundant in the fecal microbiota of the MCD–vehicle group than in the NCD–vehicle group (*p* < 0.01), whereas *Lactobacillus* and *Ruminococacceae_UCG_014* were more abundant in the NCD–vehicle group (Figure 7B and Supplementary Figure 3B). Compared to the MCD–vehicle group, the MCD–*SB* group had more abundant *Faecalibaculum, Coriobacteriaceae_UCG_002*, and *Bacteroides* (*p* < 0.01) (Supplementary Figure 2B). The most abundant taxa at the family and genus levels are presented in Figures 7A,B and Supplementary Figures 3A,B.

Shannon and Simpson’s diversity indices, which estimate the alpha-diversity of the intestinal microbiota, showed that both diversity indices were significantly lower in the MCD–vehicle group; interestingly, both diversity indices were reserved in the MCD–*SB* group, indicating that *SB* administration may maintain the diversity of intestinal microbiota to the same level as the control group (Figure 7C and Supplementary Figure 3C). The UniFrac Weighted principal coordinate analysis (PCoA) and principal component analysis (PCA) plots describe the structural differences between bacterial communities, including the phylogeny and abundance distribution. On PCoA and PCA plots, both MCD groups were separated significantly from the NCD groups with or without *SB*. In addition, the MCD–*SB* group was markedly separated from the MCD–vehicle group (Figure 7D and Supplementary Figure 3D). These results indicate that the *SB* supplement enriched the diversity and changed the gut microbial configuration (Supplementary Figure 3C). The linear discriminant analysis (LDA) effect size (LEfSe) method was used to identify taxa with statistical significance in MCD groups with or without *SB*. For those with an LDA score > 4.0, the MCD–vehicle mice showed increased *Faecalibaculum*, *Coriobateriaceae_UCG_002*, and *Bacteroides*, *Mucispirillum*, *Blautia* and *Ruminiclostridium_9*; reduced *Akkermansia*, *Parabacteroides*, and *Turicibacter* (Figure 7E).

Here, we concluded that the *SB* supplement changed the bacterial taxa in the intestine. The MCD diet could reduce the bacterial diversity in the intestine, which the *SB* supplement could restore.

### The Methionine-Choline Deficient Diet Altered the Fungal Composition in the Intestine of the Mice by Increasing *Pichia* and *Trichosporon*

To examine the effects of the MCD diet and *SB* gavage on the intestinal mycobiome of mice, mouse fecal DNA was used, and metagenomic sequencing targeting the eukaryote 18S rRNA ITS1/ITS2 region was performed.

**FIGURE 8:**
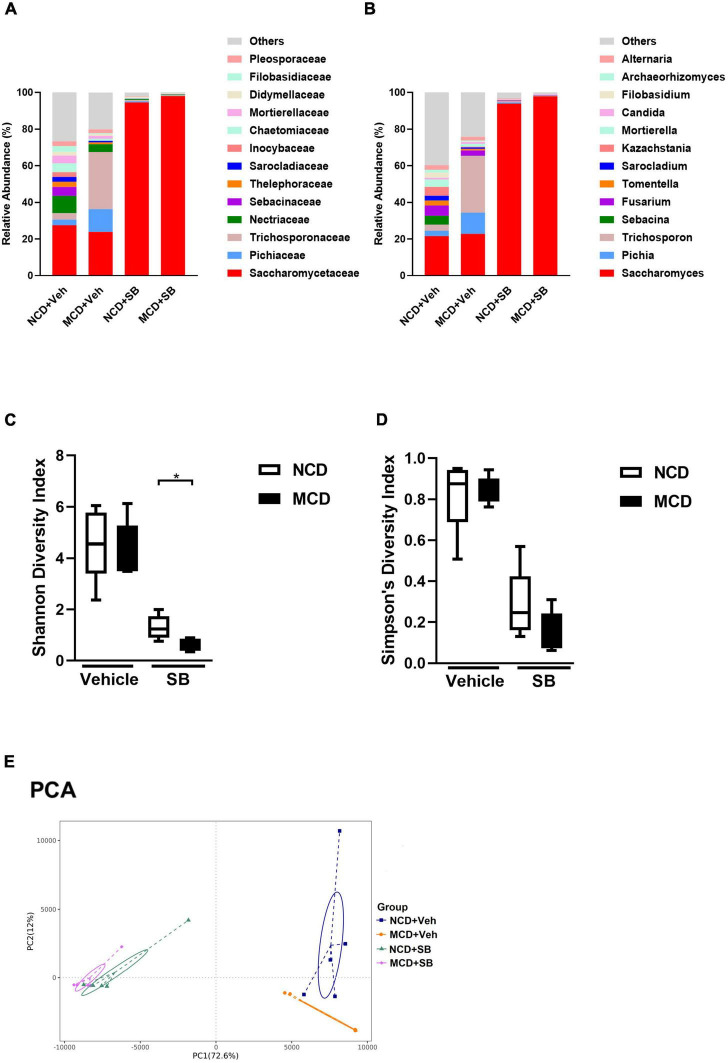
The MCD diet altered the fungal composition in the intestine of mice. **(A)** Topmost abundant taxa at the family and **(B)** genus level, MCD diet feeding showed increased *Pichia* and *Trichosporon*. Alpha diversity is represented by Shannon **(C)** and Simpson’s **(D)** diversity index. **(E)** Beta diversity presented by PCA. Each symbol represents one sample (*n* = 4–6 per group).

Both *SB* gavage groups showed a very high percentage of Saccharomyces (93.9% in the NCD group and 97.9% in the MCD group), indicating that we successfully gavaged these mice; however, a very high Saccharomyces percentage made the change of others insignificant (Figures 8A,B). MCD feeding did change the composition of intestinal mycobiome. In terms of the genus level, MCD feeding significantly increased *Pichia* and *Trichosporon* (Figure 8B and Supplementary Figure 4A). As far as alpha diversity was concerned, both Shannon and Simpson’s diversity indices were not significant between NCD–vehicle and MCD–vehicle groups. Due to the very high *Saccharomyces* percentage in both groups gavaged by *SB*, the alpha diversity of these two groups was markedly lower than that of the vehicle gavage groups. In the two *SB* gavage groups, the Shannon diversity index of the NCD–*SB* group was significantly higher than the MCD–*SB* group (Figure 8C). However, it is not significant in Simpson’s diversity index (Figure 8D). For beta diversity, the UniFrac Weighted PCoA plot of the MCD–vehicle group was separated from both NCD groups. The NCD–*SB* group and MCD–*SB* group were in the same quadrant owing to the very high percentage of Saccharomyces (Supplementary Figure 4D). Additionally, the PCA plot showed the same characteristics as PCoA (Figure 8E). In this section, we found that MCD diet feeding did change the composition of mouse intestinal fungi, characterized by increased *Pichia* and *Trichosporon.* Interestingly, both *Pichia* and *Trichosporon* belong to yeast, implying that yeast could thrive in the intestine under even worse conditions.

#### Conclusion

*SB* ameliorated MCD diet-caused hepatic steatohepatitis and subsequent fibrosis by restoring the intestinal architecture, reducing intestinal inflammation, and maintaining the gut barrier. Increased intestinal microbiome diversity and changing the configuration of the microbiota to favorable species are likely to be the reasons for the beneficial effect of the *SB* supplement.

## Discussion

Non-alcoholic fatty liver disease (NAFLD) and NASH have been a burden on health care in the modern world. Many pieces of research and clinical trials targeting different mechanisms of the disease have been carried out, but few have been applied to the clinical setting. Our report focused on the gut-liver axis and tried to test the effect of a commercially available dietary supplement yeast, *Saccharomyces Boulardii* (*SB*), on NAFLD/NASH. Administrating a probiotic yeast, *SB*, can attenuate MCD diet-related non-alcoholic fatty liver disease (NAFLD) and non-alcoholic steatohepatitis (NASH) in mice. Furthermore, NASH-related hepatic fibrosis was also ameliorated. We found that the *SB* supplement could significantly improve liver function and lipid accumulation. Our findings point to a possible strategy for the prevention of NASH and subsequent fibrosis—simply taking a dietary supplement yeast, *SB*. It has been widely used as a dietary supplement for irritable bowel syndrome in the Western world, with few safety concerns. Thus, our findings could easily be applied to humans in clinical settings.

Our findings indicated that hepatic inflammation was caused globally in the MCD diet-fed mice and was markedly suppressed in the MCD–*SB* diet-fed mice. Furthermore, less activation of Kupffer cells was found in SB-fed mice. Along with less hepatic inflammation, the MCD–*SB* diet-fed mice showed significantly less liver fibrosis. *SB* administration could reduce inflammation of the liver in all pathways and decrease the degree of subsequent liver fibrosis. Since proinflammatory cytokines involved in all the pathways were suppressed, we postulated that the key to the mechanism of liver protection should be upstream of the liver—the gut.

In this study, the MCD diet-fed mice showed shortening of the small intestines and the intestinal villi and narrowing of the intestinal crypts, which is consistent with a previous report by Schneider et al. (2019). The report by Schneider et al. showed only a shortening of the intestinal length and villi in the MCD diet-fed mice, whereas the gene expression of proinflammatory cytokines in both ileum and colon remained unchanged. In our cohort, the MCD diet-fed mice on the *SB* supplement had significantly longer small intestines and intestinal villi, as well as wider intestinal crypts. To evaluate the possibility of intestinal inflammation, gene expression profiles were checked in the proximal, middle, and distal small intestine as well as the colon. We found that *Il1-*β was caused by the MCD diet in the small intestine and colon and was significantly suppressed by *SB* supplementation. *Tnf*-α was also caused by the MCD diet and was suppressed in the proximal and middle small intestines and the colon by SB supplementation. As far as the chemotactic cytokine, *CCL2*, was concerned, it was not induced in the proximal small intestine but in the middle, distal small intestine, and the colon. *SB* supplementation successfully suppressed *CCL2* expression in the middle and distal small intestine but not in the proximal small intestine and the colon. Our findings indicated that acute-phase reaction cytokine gene expressions (*Il1-*β and *Tnf*-α) were induced in the entire intestines, which could be suppressed by the *SB* treatment, except for *Tnf*-α in the distal small intestine. For chemotactic cytokine, *CCL2*, *SB* treatment only affected the middle and distal small intestines. A recent report by Matthews et al. also showed that the MCD diet-fed mice exhibited mucosal and submucosal inflammatory responses with more intestinal proinflammatory signaling and cytokine expression (Matthews et al., 2021). They found that MCD-mimicking media does not alter the proinflammatory responses of intestinal epithelial cells but enhances macrophage proinflammatory responses. Their results suggest that macrophages, rather than intestinal epithelial cells, play an important role in the underlying mechanism of MCD diet-caused intestinal inflammation. Although the *SB* treatment in the MCD diet-fed mice could suppress most pro-inflammatory cytokine expression in the intestine, the cytokine expressions were consistently higher than their NCD–fed counterparts, suggesting that *SB* treatment only lowered the inflammation but not returned to the baseline. It suggested that *SB* treatment could improve a substantial portion of the phenotype of the MCD diet-caused intestinal injury and reduce the inflammation in the intestine.

The mechanism of MCD diet-related NASH was reported by Rinella et al. (2008). They concluded that the MCD diet increases fatty acid uptake and decreases VLDL secretion, promoting intrahepatic lipid accumulation and steatosis. Luther et al. (2015) reported a meta-analysis concerning the association of human NASH and intestinal permeability followed by the MCD diet mouse model to elucidate the mechanism. They concluded that human NASH was associated with increased intestinal permeability. A subsequent study on mice by the same investigators showed that an early phase of hepatic injury and inflammation contributes to altered intestinal permeability. A cell study done in the same report concluded that the MCD diet alone did not alter intestinal permeability, suggesting the permeability change was from a cross-talk between the liver and gut. In our study, we found that fecal albumin levels were increased in the MCD–vehicle mice and returned to baseline in the *SB* and MCD diet-fed mice, indicating that the *SB* treatment could ameliorate gut leakage. In addition, the LPS levels were elevated in the MCD–vehicle group and were tempered with in the MCD–*SB* group. Our findings suggest that *SB* protects against MCD diet-related NASH by improving gut permeability, decreasing endotoxin translocation, and decreasing inflammation in the intestine.

Since we infused a living yeast, *SB*, into the intestines of the mice, the change in the intestinal microbiota may have played a role in the effect. Our results of the OTU analysis revealed that the microbial taxa of NCD–fed mice were not altered after *SB* gavage. Both groups showed *Lactobacillus* and *Ruminococacceae_UCG_014* dominance. *Lactobacillus* is a well-known probiotic that can synthesize acetate and lactate, which could be health-promoting. *Ruminococacceae spp.* could degrade and convert complex polysaccharides into a variety of nutrients for their hosts, making them beneficial (la Reau and Suen, 2018). This result implies that adding *SB* to the intestine does not alter the microbial configuration in physiological conditions. In contrast, the MCD–vehicle mice showed significantly more abundant *Akkermansia*, *Parabacteroides*, and *Turicibacter* than their NCD counterparts. This implies that MCD diet feeding did alter the microbial configuration in the intestine. There is only one known species of the *Akkermansia* genus: *Akkermansia muciniphila.* Studies on rodents have indicated that *Akkermansia muciniphila* in the intestinal tract may reduce the risk of obesity and diabetes (Everard et al., 2014; Yassour et al., 2016). Interestingly, these effects are compatible with the phenotype of the MCD diet-fed mice. *Parabacteroides* is a gram-negative, anaerobic genus, while *Turicibacter* is a genus in the Firmicutes phylum of bacteria that has most commonly been found in the guts of animals. The role of *Parabacteroides* and *Turicibacter* in the intestine is, however, unknown.

Regarding the MCD diet-fed mice with *SB* supplement, the most dominant microbial taxon became *Faecalibaculum*, which is different from both the MCD–vehicle and control mice on normal chow. *Faecalibaculum* is a genus of gram-positive, anerobic bacteria. Its only known species is *Faecalibaculum rodentium*, which could produce both butyrate and lactate. It has been reported that *Faecalibaculum rodentium* or its metabolic products reduce tumor growth (Zagato et al., 2020).

The aforementioned results revealed that the MCD diet feeding did change the intestinal microbiome. Adding *SB* altered the intestinal microbial configuration of MCD diet-fed mice but did not restore the microbial taxa to their normal chow control counterpart. However, increased *Faecalibaculum rodentium* levels in the MCD–*SB* group may contribute to the protective effect by producing beneficial metabolic products such as butyrate and lactate.

The alpha diversity represented by Shannon and Simpson’s diversity indices showed that MCD diet feeding significantly reduced the diversity of the species and was preserved in the *SB* supplement group. A review article published by Mosca et al. (2016) pointed out that increased intestinal microbiome diversity is beneficial to the intestinal ecosystem and human health. The beta diversity showed that the clusters between the MCD–vehicle and MCD–SB groups were significantly separated, suggesting that the *SB* supplement changed the species configuration of the microbiota. Increased intestinal microbial diversity and the changed configuration of the microbiota to favorable species are likely to be the reasons for the beneficial effect of the *SB* supplement.

Eukaryote 18S rRNA ITS1/ITS2 region metagenomic sequencing of mouse fecal DNA showed increased *Pichia* and *Trichosporon* among MCD–vehicle mice. *Pichia* is a genus of yeasts in the family *Saccharomycetaceae*. *Pichia* is a genus with more than 100 species. *Pichia pastoris*, for example, is a well-known genetic and experimental model organism that is widely used in biological research. *Trichosporon* is also a yeast with hyphae. It is a well-known cause of an unpleasant but harmless hair condition known as white Piedra (Magalhães et al., 2008). *Trichosporon* could be an opportunistic pathogen that causes serious infections (Marty et al., 2003) in immunocompromised individuals. Interestingly, these fungal genera, both yeasts with hyphae, increased after the MCD diet. We assumed that MCD diet feeding might create a worse environment in the intestine, allowing only durable fungi such as yeast-like organisms to survive. Both *SB* gavage groups showed a very high percentage of *Saccharomyces*, which implied that we successfully gavaged the mice; however, this also made the changes of other fungi insignificant.

In this study, we demonstrated that mice on the MCD diet exhibited increased intestinal inflammation and permeability. Through the leaky gut, endotoxins could enter hepatic sinusoids via the portal circulation, causing steatosis and inflammation of the liver. The *SB* supplement reduced intestinal inflammation and changed the configuration of the intestinal microbiome to favorable species. These intestinal protective effects of *SB* may prevent MCD diet-related leaky gut, ameliorate subsequent hepatic steatohepatitis, and fibrosis through the gut-liver axis (Schnabl and Brenner, 2014).

Nevertheless, this study is not without some limitations. First, although the MCD diet model is a well-known NASH animal model, it is not entirely representative of human NASH because of the absence of features such as obesity and insulin resistance. Further study is needed to validate the correlation. Second, in our model, the *SB* supplement and MCD diet feeding were started on the same day. Hence, the beneficial effect could be protective rather than restorative. Lastly, although our results showed that the *SB* supplement changed the intestinal microbiome and alleviated leaky gut and subsequent hepatic steatohepatitis, whether the change in the microbiome contributed to the beneficial effect requires further investigation.

Our study demonstrates the beneficial effect of *SB* on NAFLD/NASH. *SB* is a commercially available dietary supplement with a good safety profile, making it easy to translate our findings into clinical practice. Our results provide insight into the prevention of NAFLD/NASH.

## Data Availability Statement

The data presented in this study are deposited in the NCBI repository, accession number PRJNA817530.

## Ethics Statement

The animal study was reviewed and approved by Institutional Animal Care and Use Committee of Taipei Veterans General Hospital, Taiwan (IACUC number: 2018–012).

## Author Contributions

A-MY and K-CL: study concept and design and drafting of the manuscript. A-MY, K-CL, and H-JS: acquisition of data. A-MY, K-CL, C-YL, S-HL, G-DS, H-CL, and M-CH: analysis and interpretation of data. All authors contributed to the article and approved the submitted version.

## Conflict of Interest

The authors declare that the research was conducted in the absence of any commercial or financial relationships that could be construed as a potential conflict of interest.

## Publisher’s Note

All claims expressed in this article are solely those of the authors and do not necessarily represent those of their affiliated organizations, or those of the publisher, the editors and the reviewers. Any product that may be evaluated in this article, or claim that may be made by its manufacturer, is not guaranteed or endorsed by the publisher.
