# Supplementary material for: Saccharomyces Boulardii Ameliorates Non-alcoholic Steatohepatitis in Mice Induced by a Methionine-Choline-Deficient Diet Through Gut-Liver Axis
Source: Front Microbiol. 2022 Jun 23;13:887728. doi: 10.3389/fmicb.2022.887728 (PMC9260146; doi:10.3389/fmicb.2022.887728)
Supplement: Supplementary file 1 [file Data_Sheet_1.docx]

**Supp. Fig 1**

(A) Liver/body weight ratio is significantly lower in MCD-fed mice. After *SB* gavage, the liver/body weight ratio becomes insignificant between control and MCD-fed mice. (B) The body weight was not different between MCD or NCD mice gavage *SB* or not.

**Supp. Fig 2**

Comparison of the abundance of intestinal microbial taxa between MCD-vehicle group and NCD-vehicle group, MCD-vehicle group and MCD-*SB* group. At family level (A) and genus level (B).

**Supp. Fig 3**

Heatmap of intestinal microbial taxa at family level (A) and genus level (B). Alpha diversity represented by Simpson’s diversity index (C). PCoA plot of the microbiota based on weighted UniFrac metric (D).

**Suppl. Fig 4**

The abundance of fungal taxa between MCD-vehicle and NCD-vehicle group (A). Heatmap of intestinal fungal taxa at family level (B) and genus level (C). PCoA plot of the fungal taxa based on weighted UniFrac metric (D).

**Supplement Table 1:** Real-time qPCR Primers.

| Mouse |  |  |  |  |  |  |
| --- | --- | --- | --- | --- | --- | --- |
| α-SMA | forward | 5′-GTT CAG TGG TGC CTC TGT CA-3′ | | | | |
|  | reverse | 5′-ACT GGG ACG ACA TGG AAA AG-3′ | | | | |
| TGF-β1 | forward | 5′-GTG GAA ATC AAC GGG ATC AG-3′ | | | | |
|  | reverse | 5′-ACT TCC AAC CCA GGT CCT TC-3′ | | | | |
| Collagen 1a1 | forward | 5′-GAG CGG AGA GTA CTG GAT CG -3′ | | | | |
|  | reverse | 5′- GCT TCT TTT CCT TGG GGT TC-3′ | | | | |
| MMP2 | forward | 5′-CTG ATA ACC TGG ATG CCG TCG T -3′ | | | | |
|  | reverse | 5′-TGC TTC CAA ACT TCA CGC TCT T -3′ | | | | |
| TIMP1 | forward | 5′-TGG GGA ACC CAT GAA TTT AG-3′ | | | | |
|  | reverse | 5′-ATC TGG CAT CCT CTT GTT GC A-3′ | | | | |
| IL-6 | forward | 5′-CTC TGG GAA ATC GTG GAA AT-3′ | | | | |
|  | reverse | 5′-CCA GTT TGG TAG CAT CCA TC A-3′ | | | | |
| IL-1β | forward | 5′-GAA CCA AGC AAC GAC AAA-3′ | | | | |
|  | reverse | 5′-GCA GAC TCA AAC TCC ACT-3′ | | | | |
| CCL2 | forward | 5′-ATT GGG ATC ATC TTG CTG GT-3′ | | | | |
|  | reverse | 5′-CCT GCT GTT CAC AGT TGC C-3′ | | | | |
| IFN-γ | forward | 5′- TGA TGG CCT GAT TGT CTT TCA A -3′ | | | | |
|  | reverse | 5′- GGA TAT CTG GAG GAA CTG GCA A -3′ | | | | |
| TNF-α | forward | 5′-TGC CTA TGT CTC AGC CTC TTC-3′ | | | | |
|  | reverse | 5′-GAG GCC ATT TGG GAA CTT CT-3′ | | | | |
| F4/80 | forward | 5′-TGC CAC CTG CAC TGA CAC CAC-3′ | | | | |
|  | reverse | 5′-AGC TGC ACT TGG CTC TCC CCA-3′ | | | | |
| IL-10 | forward | 5′-ATG CTG CCT GCT CTT ACT GAC TG-3′ | | | | |
|  | reverse | 5′-CCC AAG TAA CCC TTA AAG TCC TGC-3′ | | | | |
| 18S RNA | forward | 5′-GCA ATT ATT CCC CAT GAA CG-3′ | | | | |
|  | reverse | 5′-GGC CTC ACT AAA CCA TCC AA-3′ | | | | |
| ZO-1 | forward | 5′-AGG ACA CCA AAG CAT GTG AG-3′ | | | | |
|  | reverse | 5′-GGC ATT CCT GCT GGT TAC A-3′ | | | | |
