# Supplementary figures and images for: Saccharomyces Boulardii Ameliorates Non-alcoholic Steatohepatitis in Mice Induced by a Methionine-Choline-Deficient Diet Through Gut-Liver Axis
Source: Front Microbiol. 2022 Jun 23;13:887728. doi: 10.3389/fmicb.2022.887728 (PMC9260146; doi:10.3389/fmicb.2022.887728)

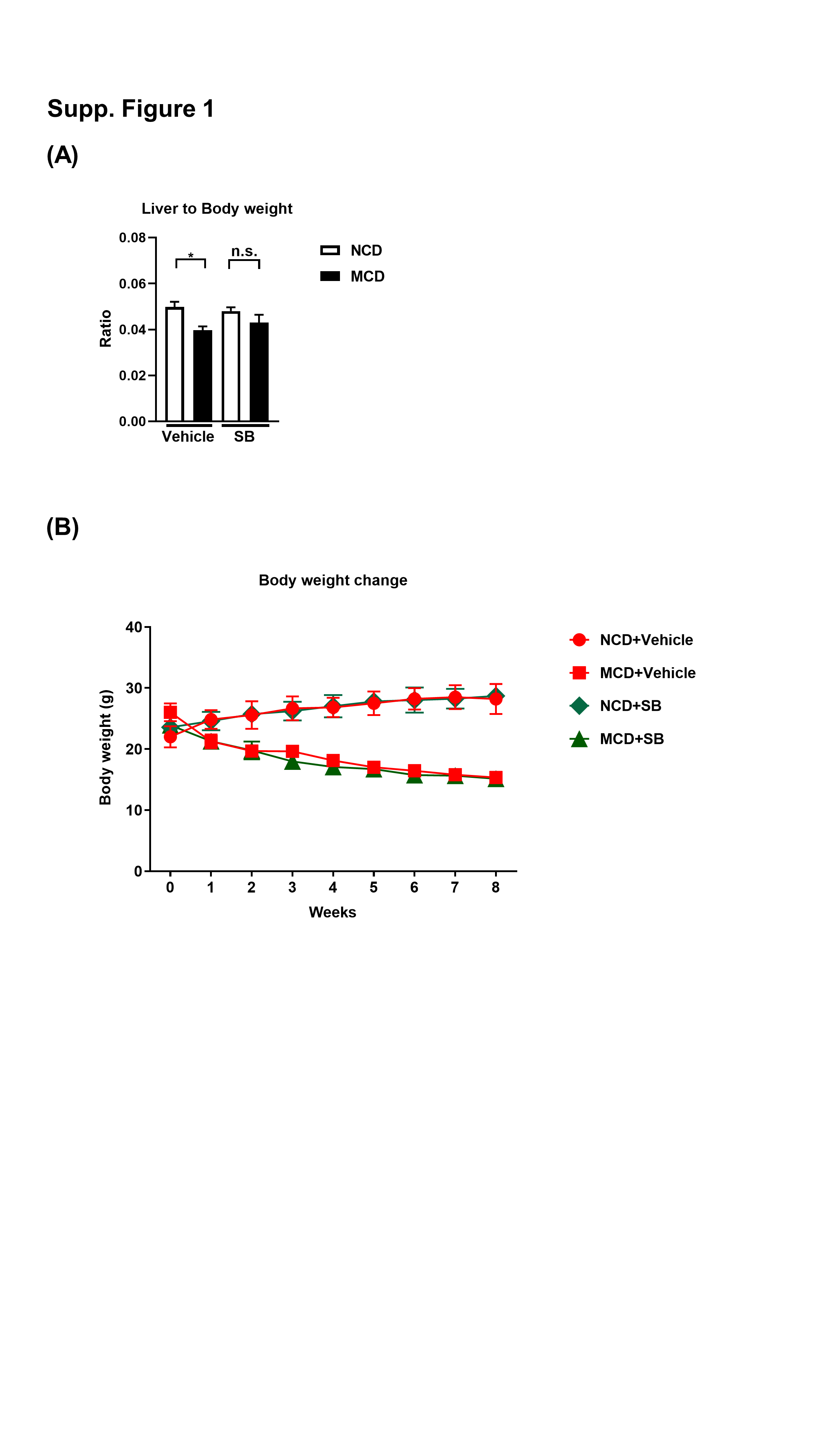

Supplement: Supplementary file 2 [file Image_1.TIF]

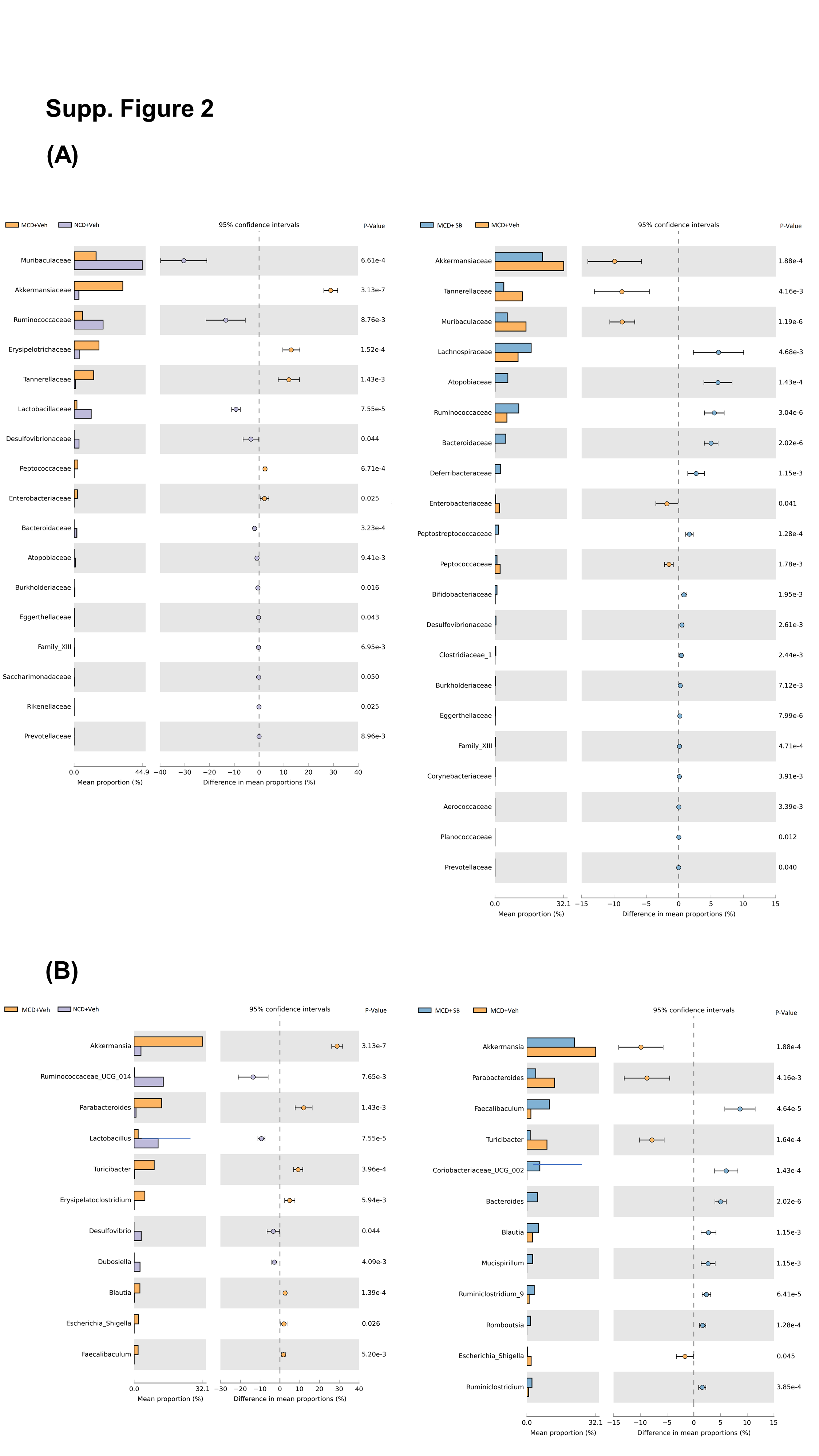

Supplement: Supplementary file 3 [file Image_2.TIF]

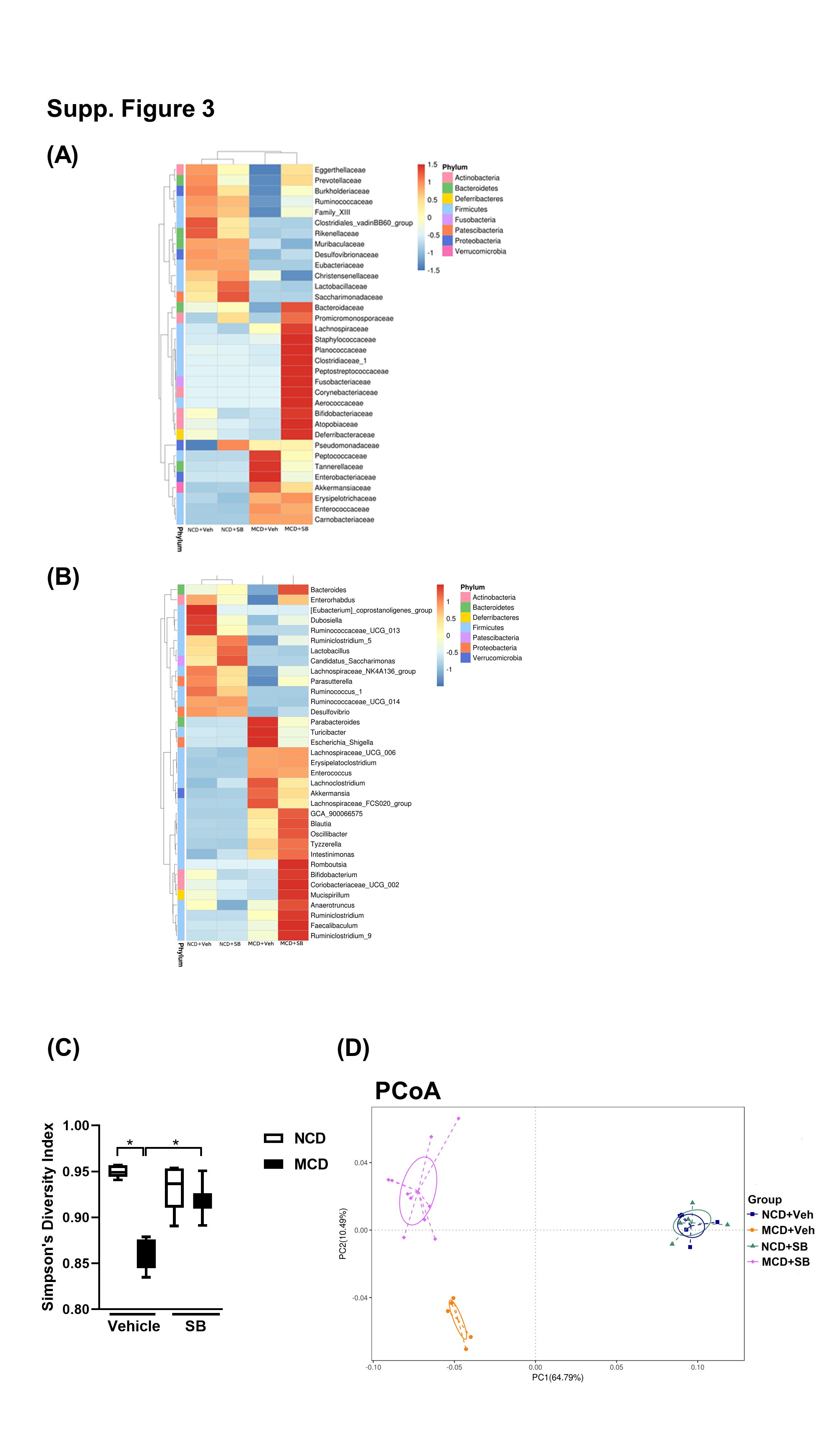

Supplement: Supplementary file 4 [file Image_3.TIF]

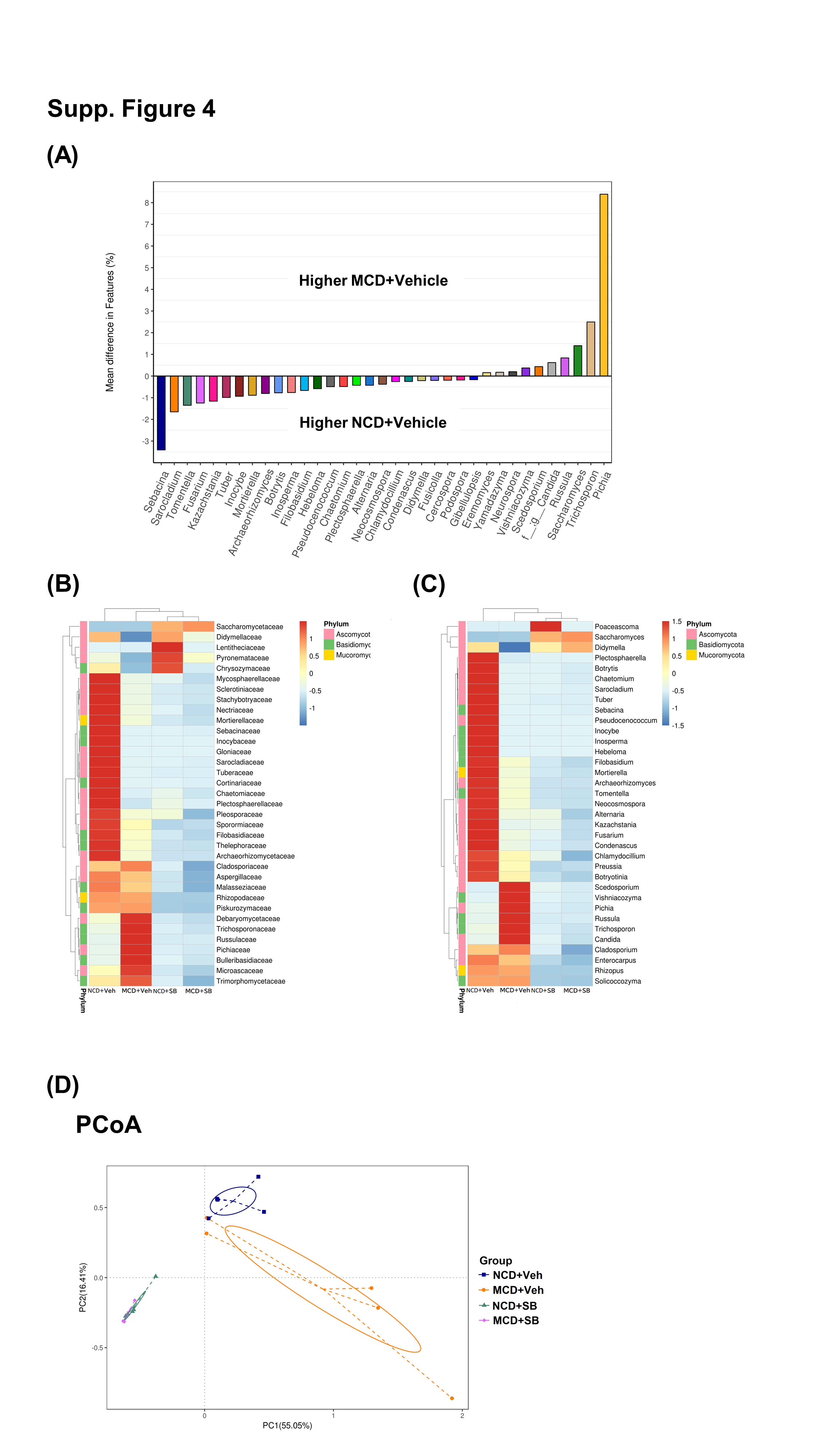

Supplement: Supplementary file 5 [file Image_4.TIF]
